# Glioblastoma Chemoresistance: The Double Play by Microenvironment and Blood-Brain Barrier

**DOI:** 10.3390/ijms19102879

**Published:** 2018-09-22

**Authors:** Martina Da Ros, Veronica De Gregorio, Anna Lisa Iorio, Laura Giunti, Milena Guidi, Maurizio de Martino, Lorenzo Genitori, Iacopo Sardi

**Affiliations:** 1 Neuro-oncology Unit, Department of Pediatric Oncology, Meyer Children’s Hospital, Florence, 50139, Italy; martina.daros@meyer.it (M.D.R.); veronicadegregorio@libero.it (V.D.G.); annalisa.iorio@meyer.it (A.L.I.); mileguidi@hotmail.it (M.G.); 2 Medical Genetics Unit, Meyer Children’s University Hospital, 50139 Florence, Italy; laura.giunti@meyer.it; 3 Director Post Graduate Pediatric School University of Florence, Director Meyer Health Campus, Florence, 50139, Italy; maurizio.demartino@unifi.it; 4 Neurosurgery Unit, Department of Neurosciences, Meyer Children’s Hospital, Florence, 50139, Italy; lorenzo.genitori@meyer.it

**Keywords:** glioblastoma, chemoresistance, microenvironment, blood-brain barrier

## Abstract

For glioblastoma, the tumor microenvironment (TME) is pivotal to support tumor progression and therapeutic resistance. TME consists of several types of stromal, endothelial and immune cells, which are recruited by cancer stem cells (CSCs) to influence CSC phenotype and behavior. TME also promotes the establishment of specific conditions such as hypoxia and acidosis, which play a critical role in glioblastoma chemoresistance, interfering with angiogenesis, apoptosis, DNA repair, oxidative stress, immune escape, expression and activity of multi-drug resistance (MDR)-related genes. Finally, the blood brain barrier (BBB), which insulates the brain microenvironment from the blood, is strongly linked to the drug-resistant phenotype of glioblastoma, being a major physical and physiological hurdle for the delivery of chemotherapy agents into the brain. Here, we review the features of the glioblastoma microenvironment, focusing on their involvement in the phenomenon of chemoresistance; we also summarize recent advances in generating systems to modulate or bypass the BBB for drug delivery into the brain. Genetic aspects associated with glioblastoma chemoresistance and current immune-based strategies, such as checkpoint inhibitor therapy, are described too.

## 1. Introduction

Alongside the definition of cancer as a genetic disease, in recent years, the key role of the relation between tumor epithelium and tissue microenvironment in the tumorigenesis has been outlined: the dynamic interaction between microenvironment and cancer cells promotes the growth, proliferation and protection of the tumor from immune surveillance and therapy [1].

It has emerged that tumor progression takes place by a Darwinian selection of the favorite clones [2], and mutator phenotypes allow greater adaptations to the microenvironment [3]. Therefore, tumor cells influence the microenvironment, which in turn selects the fittest clones. In response to tumor growth, microenvironment changes continuously [4].

Glioblastoma represents a prototype of tumor in which the remarkable capability of communication with the microenvironment and the complex intra- and inter-tumor heterogeneity play a leading role in tumor malignancy, invasiveness and therapy failure. The interaction of tumor-glia cells is also linked with the chemoresistant phenotype of glioblastoma [5]; in particular, the hypoxic microenvironment, a common feature of this type of cancer, is strongly associated with radio- and chemoresistance by modulating different mechanisms, such as apoptosis and angiogenesis [6], expression of ATP-binding cassette (ABC) proteins [7], and functions of glioblastoma stem cells (GSCs) [8].

Notably, cancer cells can acquire a resistant phenotype in response to therapy, or they can be intrinsically resistant due to genetic aberrations underlying tumor development [9]. In both cases, the altered expression of multi-drug resistance (MDR)-related genes is associated with a reduced responsiveness to therapy [10].

In addition, poor permeability of anticancer drugs through the BBB and their inability to reach the tumor mass at a therapeutic concentration decrease the effectiveness, thus contributing to treatment failure [11].

Finally, the immunosuppressive feature of the tumor microenvironment (TME) allows cancer cells to sidestep immune surveillance, thus progressing [12].

This review provides an overview on how microenvironment affects glioblastoma response to therapy, on the direct pharmacological modulation of BBB drugs’ permeability, on involvement of genetic pathways in the chemoresistant phenotype of glioblastoma, and on how the brain’s microenvironment could offer the opportunity to implement treatment of glioblastoma patients.

## 2. Chemoresistance Due to Changes in TME

Several mechanisms and biological processes are responsible for the poor response of glioblastoma to antineoplastic treatments. Interestingly, the TME, which consists of cells, specific factors, and conditions, plays a key role in glioblastoma chemoresistance. 

Glioblastoma is a very heterogeneous tumor surrounded and sustained by a heterogeneous microenvironment able to promote tumor cell growth and to select the most aggressive cancer cells [8,13]. In many types of cancer, including glioblastoma, groups of cells named CSCs have been identified. CSCs are multipotent and possess tumor initiation and self-renewal capacity; in addition, these cells are involved in radio- and chemoresistance, cancer aggressiveness and recurrence, invasion and metastasis [7,14,15]. The TME, interacting with CSCs and supporting their phenotype and other biological mechanisms associated with cancer, shows an important protective action and promotes tumor progression and therapeutic resistance [8,13].

The glioblastoma microenvironment harbors different types of cells; in particular, stromal cells, endothelial and immune cells sustain tumor development and are especially important for chemoresistance. This variety of cells, in combination with the extracellular matrix (ECM), cytokines, growth factors and specific conditions, such as hypoxia and acidosis [14,16], constitutes the glioblastoma microenvironment. All these key players are differently distributed in glioblastoma, delineating a variable microenvironment within the same tumor.

Better comprehension of TME could be useful for the modulation of existing therapeutic treatments or the development of new therapies against TME and the interactions between CSCs and TME, in order to reduce cancer aggressiveness, preventing tumor progression and patient relapse.

### 2.1. Endothelial Cells

Concerning endothelial cells and TME, it is known that glioblastoma is characterized by aberrant and disorganized vascular morphology and extensive angiogenesis [17,18,19], mainly associated with high expression of vascular endothelial growth factor (VEGF), an essential regulator of angiogenesis, and other proangiogenic cytokines and chemokines. Vascular alterations represent an obstacle for the delivery of antineoplastic therapies, playing a critical role in glioblastoma chemoresistance and, at the same time, interfering with oxygen and nutrient supply, promoting hypoxia, acidosis and nutrient deprivation. In particular, hypoxia induces the recruitment of immune cells that, with their proangiogenic properties, support and amplify tumor vasculature expansion [16]. Moreover, VEGF affects adhesive tumor properties, interfering with the mechanisms of cellular adhesion, diapedesis and immune cell infiltration in glioblastoma [16].

CSCs promote tumor angiogenesis; in turn, endothelial cells are able to influence CSC biology and their chemoresistance through the secretion of specific factors, such as stromal cell-derived factor-1α, nitric oxide, angiopoietins, serpins, and the involvement of specific pathways, including Notch, SHH–HEDGEHOG, ABCB1–ABCG2 and ephrins pathways [14,15].

Finally, Fianco et al. [20], using in vitro and in vivo glioblastoma models, highlighted the link between inflammation, angiogenesis and chemoresistance. The authors showed that in an inflammatory microenvironment, Caspase 8, a protein implicated in apoptosis, promotes NF-κB transcription factor activation with consequent increase of VEGF, IL-6, IL-8, IL-1β and MCP-1 secretion, enhancing neovascularization and resistance to temozolomide (TMZ), an alkylating agent widely used in glioblastoma treatment.

### 2.2. Tumor-Associated Immunosuppressive Cells

Glioblastoma is characterized by an immunosuppressive microenvironment; in particular, CSCs are involved in the recruitment of immunosuppressive cells into the TME [15] by the secretion of cytokines and chemokines; in turn, tumor-associated immunosuppressive cells support CSC phenotypes and chemoresistance, evasion from host immune surveillance and invasion. The knowledge of TME could be very useful for the development of immune-targeted therapies. 

The main tumor-associated immunosuppressive cells are tumor-associated macrophages (TAMs), myeloid-derived suppressor cells (MDSCs), T-regulatory (Treg) cells, and natural killer (NK) cells.

TAMs are grouped into two different immune phenotypes: the M1-polarized subtype, which is pro-inflammatory and antitumoral; and the M2-polarized subtype, which is anti-inflammatory and protumoral. Most of the macrophages recruited into TME become M2 subtype and induce T cell anergy, secrete ECM components and stimulate angiogenesis [15]. In some cancers, it is well documented that TAMs promote CSC phenotype and growth [15]; moreover, TAMs seem to induce modification of stromal and blood vessel architecture and enhance tumorigenicity and drug resistance [15,16,21].

MDSCs represent a population of myeloid-originated progenitor cells; they are involved in the inhibition of T cell proliferation and activity through the secretion of several substances, including inducible nitric oxide synthase, reactive oxygen species (ROS), cyclooxygenase-2 and transforming growth factor-β [15,22]; these cells appear abundantly in TME of glioblastoma [23]. In ovarian, breast and pancreatic cancer, MDSCs appear to enhance CSC properties and tumor progression [15].

Treg cells represent another cell subpopulation recruited in TME and involved in immunosuppression and regulation of CSC phenotypes by the secretion of some cytokines, such as IL-10, IL-35 and TGF-β [15,24]. In different types of cancer, including gastric, esophageal, pancreatic, liver and breast cancer [15,25,26,27,28], Treg cells have been associated with poor prognosis.

Finally, NK cells are cytotoxic lymphocytes of innate immune system able to exert a cytotoxic activity; in melanoma and glioblastoma, CSCs appear resistant to NK cells [15].

Other stromal cells hosted by TME are represented by cancer-associated fibroblasts (CAFs) [15,29,30] and neutrophils. CAFs comprise a heterogeneous and activated cell population that increase the secretion of growth factors, enzymes and components of the ECM and appear to be involved in tumor growth and in maintenance of CSC stemness. Trylcova et al. showed that CAFs are able to stimulate growth and migration of glioma cells, promoting cancer aggressiveness and a reduction of the response to therapeutic treatments [31]. Concerning neutrophils, they are involved in the regulation of CSCs [15], and in some tumors play a role in the resistance to antiangiogenic therapy [16]. Interestingly, in glioblastoma patients, an increase of neutrophils seems associated with a poor prognosis [23,32].

### 2.3. ECM

ECM is a really important component of TME; it constitutes the basement membrane and interstitial matrix and consists of glycosaminoglycans, glycoproteins and proteoglycans [15,16]. It is not only an extracellular scaffold, but also a dynamic compartment where components are continuously deposited, degraded or remodeled. This remodeling process appears critical for tissue architecture and influences some biological mechanisms. Indeed, during tumorigenesis, an increase in collagen content is common [15,33], as well as an overexpression of other ECM components. Moreover, ECM alterations contribute to the deregulation of cell proliferation, differentiation, death and invasion [34].

ECM supports CSCs and appears to be involved in tumor progression and chemoresistance. It provides receptors able to anchor CSCs in the niche and secretes growth factors that lead to CSC proliferation; in glioblastoma, CSC growth results are enhanced by ECM protein laminin alfa-2 [15,35], while integrin alfa-6 plays a role in CSC self-renewal regulation [14,36].

Interestingly, glioblastoma tissue appears stiffer compared to non-tumoral tissue [16,37]. Matrix stiffness, in combination with an increase in fluid pressure, cell compression and an increase of tumor cellular contractility, promotes glioblastoma stiffness [15,16]. All these alterations interfere with vessel integrity and could be a significant obstacle to the recruitment of inflammatory cells and the delivery of macromolecules, including chemotherapeutic agents. Moreover, matrix stiffness influences CSC plasticity regulation and CSC marker expression [15,38,39].

Finally, different molecules in the ECM can modify the recruitment of infiltrating immune cells in TME [16,40] and influence response to chemotherapy [40]. Concerning soluble factors, recently, galectin-1, a β-galactoside-binding lectin, has been described as an innovative target in glioblastoma TME for its possible involvement in therapy resistance [16], especially resistance to TMZ, tumor growth, angiogenesis, invasion and immune evasion.

### 2.4. Hypoxia

TME plays an essential role in tumor behavior, not only through the involvement of specific cell types, but also through the establishment of specific conditions.

Glioblastoma is characterized by low tumor oxygenation, a phenomenon named hypoxia, which is mainly the result of an increase of cancer cell proliferation that overcomes the limit of the blood supply [15].

Hypoxia contributes to the regulation of CSC properties, promoting a more aggressive tumor phenotype; it enhances CSC maintenance, chemoresistance and the recruitment of other cells that support tumor growth [7,15].

Interestingly, hypoxia regulates many genes involved in cancer through the activation of the transcriptional activity of proteins named hypoxia-inducible factors (HIFs). These genes are responsible for different tumoral mechanisms such as angiogenesis, survival, resistance to therapies, genomic instability, invasion and metastasis [7,15,41]. Some of these genes influence metabolism too; in particular, hypoxia promotes glucose uptake and its conversion to lactate [7,41].

Some studies have described that hypoxia increases the expression of CSCs markers in breast and prostate cancer cell lines [15,42,43]; in addition, it promotes resistance to drugs that usually target cancer cells in a proliferative state, maintaining CSCs in a quiescent state [15]. In glioblastoma in vitro models, Ahmed et al. showed, under hypoxic conditions, an increase of CD133 expression and an enhancement of resistance to cisplatin, TMZ and etoposide [44].

Hypoxia also confers radioresistance; indeed, in the absence of oxygen, there is a decrease of oxygen free radical formation associated with radiation, and consequently a reduction of DNA damage [8,13].

Another candidate mechanism through which hypoxia promotes glioblastoma chemoresistance is the inhibition of pro-apoptotic pathways. For instance, under hypoxic conditions, the pro-apoptotic protein Bad appears to be modified, and unable to interfere with pro-survival factors [7,45]; therefore, an increase of the expression of antiapoptotic proteins, such as Livin [7,46], has been reported.

The effect of hypoxia on chemoresistance is also known to be mediated by ABC transporters [7], including P-glycoprotein (P-gp or MDR1) and Multidrug resistance-associated protein 1 (MRP1). In general, an enhancement of ABC transporter expression represents one of the major MDR mechanisms that protect cancer cells from different drugs. Nardinocchi et al. observed that a downregulation of HIF-1alfa was associated with a decrease in MDR1 transcript levels [7,47]; in another study, Chen et al. showed in T98G cells that HIF-1alfa knockdown reduced the expression of MRP1 transporter, sensitizing cells to doxorubicin (Dox) and etoposide [7,48].

Concerning other connections between hypoxia and chemoresistance, according to Rosa et al., in an in vitro model hypoxia regulates calcium-activated potassium channels that seem to enhance the aggressiveness of glioblastoma induced by low oxygenation and especially invasiveness and resistance to cisplatin [49].

Moreover, hypoxia appears involved in resistance to alkylating compounds through the activation of targets in the mammalian target of rapamycin (mTOR) pathway, in particular the N-myc downstream regulated gene 1 (*NDRG1*) [50].

### 2.5. Acidosis

In tumors, not only hypoxia, but also acidosis, and especially high lactate levels, represents a critical stress factor of TME and influences tumor behavior. Indeed, tumor cells, in order to support their high proliferation rate, show an upregulation/acceleration of glycolysis, a phenomenon named the “Warburg effect” and associated with a rapid energy supply and an increase in the conversion of glucose to lactate [51,52,53,54].

Lactate influences tumor progression, resistance to radiochemotherapy and immune escape [55,56,57]. In glioblastoma, TME acidosis is involved in disease progression and poor survival [55].

**Figure 1:**
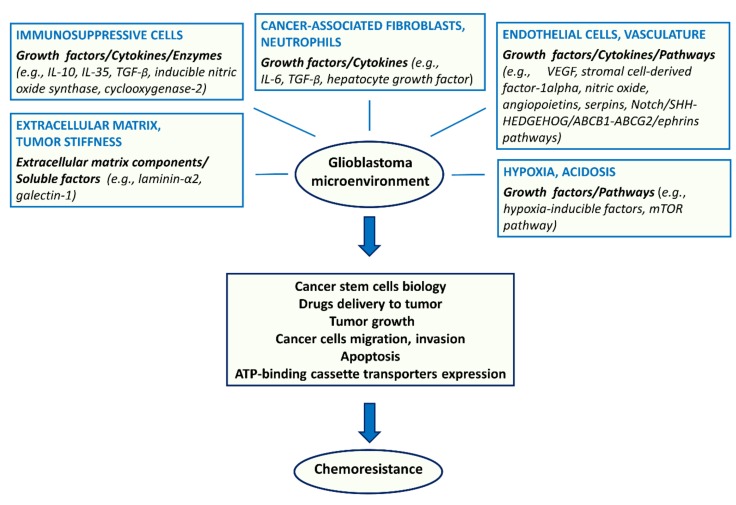
Schematic representation of the main components (endothelial cells, vasculature, immunosuppressive cells, CAFs, neutrophils, ECM, tumor stiffness, hypoxia, acidosis) and molecules of glioblastoma microenvironment involved in chemoresistance. Glioblastoma microenvironment, interacting with CSCs and influencing other mechanisms associated with cancer, promotes chemoresistance.

A schematic representation of glioblastoma microenvironment and its involvement in chemoresistance is shown in Figure 1.

In addition, some studies have shown that acidosis promotes cell motility, migration, degradation and remodeling of the ECM [55,58,59]. Furthermore, in human glioma cells, lactate appears to be involved in tumor angiogenesis and to support the expression of markers of CSCs [60,61].

It is known that exposure to acidic pH also induces a high level of autophagy, a critical biological process associated with the maintenance of CSC phenotype and resistance to treatments [51,62,63,64].

Concerning chemoresistance, acidosis promotes this phenomenon, reducing the uptake and the efficacy of different drugs, such as anthracyclines, anthraquinones and vinca alkaloids [51]. Moreover, it neutralizes the ROS formation associated with radiotherapy [55], inhibits radiation-induced apoptosis [51], enhances the pump activity of P-gp [51] and promotes a low proliferative rate of tumor cells, making them less sensitive to chemotherapy [51,65,66].

Finally, acidosis promotes immune escape by inactivating proliferation, tumor infiltration and cytokine release of T cells [54,55,58], blocking monocytes [55], inhibiting the cytotoxic activity of NK and CD8+ T cells and enhancing the MDSCs activity [54].

## 3. BBB Pharmacological Modulation for Treatment of Human Glioblastoma

BBB is the term used to describe the unique properties of the microvasculature of the central nervous system (CNS). The BBB is composed of endothelial cells, astrocyte end-feet and pericytes [67]; this anatomical and physiological structure separates the brain from the circulatory system, protecting it from harmful agents, regulating the transport of essential molecules, and maintaining a stable microenvironment.

The BBB functions are dynamic (not fixed). In fact, under physiological conditions, BBB is able to respond to a variety of regulatory signals from both the blood and brain sides, but its activities can be significantly disturbed under pathological conditions.

Glioblastoma development alters BBB integrity, but surgical and radiographic information have evidenced that all glioblastoma patients present brain regions with an intact BBB sufficient to prevent drug distribution to tumor cells [68,69]. The BBB, in fact, is reported to restrict the diffusion from the bloodstream into the brain parenchyma of 100% of large-molecule and 98% of small-molecule drugs by means of tight junctions between capillary endothelial cells and efflux activity of ABC transporters [70,71,72]. This evidence suggests that delivery of therapeutic agents across the BBB is essential to making significant progress in glioblastoma treatment.

**Figure 2:**
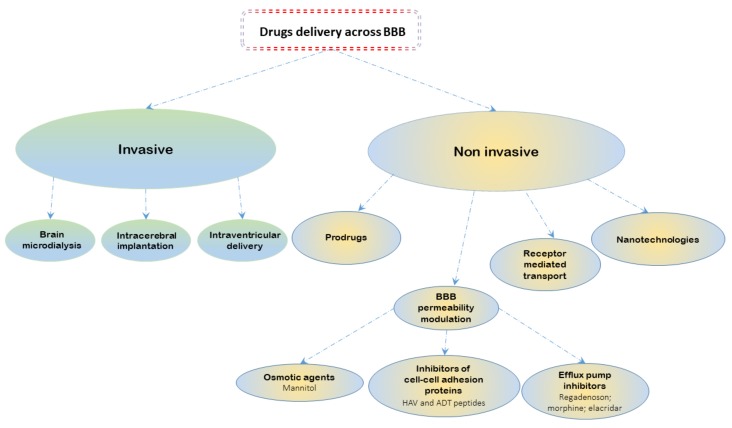
Schematic representation of the most used approaches to overcome the BBB-mediated chemoresistance.

As shown in Figure 2, different invasive methods have been tested to increase drug delivery into the brain; these methods include brain microdialysis [73], intracerebral implantation [74], and intraventricular delivery [75]. However, these approaches could cause damage to the surrounding healthy tissue and severe side effects in patients. 

Instead, various non-invasive approaches have been explored to improve drug delivery through BBB and to limit side effects (Figure 2). Several data have been reported in the literature about the use of prodrug [76,77], nanotechnologies [78,79] and receptor-mediated transport methods that modify the drugs’ structure or administration, but the most interesting and least invasive approach seems to be the direct pharmacological modulation of BBB permeability through BBB modulators such as osmotic agents and, in particular, efflux pump inhibitors.

### 3.1. Paracellular Modulation

A promising approach for delivering molecules across the BBB is via the paracellular pathway, by increasing the porosity of the tight junctions.

One successful method for enhancing paracellular delivery through the BBB is the use of hyperosmotic agents such as mannitol. The administration of osmotic agents, in fact, produces a hypertonic environment within the brain vasculature, causing shrinkage of endothelial cells and opening the tight junctions [80].

Current in vivo research indicates that the intraarterial injection of the hyperosmotic agent mannitol significantly improves BBB permeability to drugs [81,82]. However, earlier research with this hyperosmotic agent showed that its administration may increase neurotoxicity [83,84]. Despite these observations, Phase I clinical trials with patients who had malignant gliomas have reported significant radiological evidence of tumor reduction and improved progression-free survival after mannitol-mediated BBB disruption followed by treatment with bevacizumab and melphalan/carboplatin, respectively [82,85].

Also, bradykinin-like compounds (histamine, leukotrienes, bradykinin) have been reported to disrupt tight junctions by stimulating B2 receptors expressed on endothelial cells and transiently increasing cytosolic Ca2^+^ [86]. Inamura et al. reported that low-dose bradykinin selectively increased blood–tumor barrier permeability in an in vivo model of intracerebral tumors [87].

Another strategy adopted for BBB disruption is the modulation of the intercellular junctions by using inhibitors of cell-cell adhesion proteins (occludins, claudins, cadherins). Different studies have utilized HAV (His-Ala-Val) [88] and ADT (Ala-Asp-Thr) [89] peptides, derived from the extracellular domain of E-cadherin, to modulate the BBB tight junctions and improve paracellular penetration of different molecules. Ulapane et al. reported that a cyclic ADT peptide, ADTC5, enhances the delivery of functional molecules, such as 65 kDa galbumin and peptides (e.g., cIBR7), creating a short opening (longer than 10 min but shorter than 40 min) of the BBB in mouse and rat models. They also conclude that BBB modulation by cadherin peptides depends on different factors, such as the type and dose of modulator peptide, the timing of delivery between BBB modulator and the delivered molecule, and the size of the delivered molecules [90]. Another interesting study was conducted by Laksitorini et al. using different cyclic ADT peptides (ADTC1, ADTC5, ADTC6). They reported the ability of these peptides to enhance the paracellular permeation of marker molecules, measuring the change in transepithelial electrical resistance (TEER) values of MDCK (madin-darby canine kidney, a widely used BBB in vitro model) cell monolayers as a function of peptides concentrations. They also demonstrated that these cyclic peptides enhanced brain delivery of ^14^C-mannitol, ^14^C- or ^3^H-polyethylene glycols (PEG) and gadolinium-DTPA (Gd-DTPA), in an in situ rat brain perfusion model and in balb/c mouse model [91].

Interesting results have also been obtained using HAV peptides. MDCK cells pretreated with a HAV-based peptide (Ac-SHAVSS-NH2) showed an increased paracellular diffusion of mannitol and a decreased TEER value [88]. Furthermore, this peptide was able to enhance the brain delivery of 3H-daunomycin in an in situ rat perfusion model [92]. Based on this evidence, a recent study has evaluated the effect of this HAV peptide on BBB permeability in vivo, with special attention paid to the time to onset and duration of changes in cerebral vascular permeability. Administration of HAV peptide resulted in a dose-dependent increase in the accumulation of the Gd-DTPA contrast agent in all brain regions, with an approximately 2–4-fold increase in Gd-DTPA intensity in the HAV treatment group compared to control mice. The time frame for BBB disruption was also determined using MRI techniques. The increase in Gd-DTPA accumulation in the brain was rapid and transient; the effect of the HAV peptide on BBB permeability appeared within 3–6 min and ended 1 h following administration.

More interestingly, HAV peptide was able to deliver to the brain a large macromolecule paracellular marker, IRDye 800CW PEG, as well as a small molecule P-gp substrate, rhodamine 800 (R800), without any disruption in cerebral blood flow. In the case of R800, there was an approximately 2-fold increase of the small molecule accumulation in the brain following HAV peptide exposure; data comparable to the increases in brain accumulation of R800 were observed following treatment with the P-gp inhibitor elacridar [93].

P-gp activity is the main cause of the MDR phenotype in glioblastoma. Numerous molecularly targeted agents and anticancer drugs have demonstrated substrate affinity to this efflux protein, which limits their ability to cross the BBB.

### 3.2. Transcellular Modulation

One approach to modulating the active efflux of potentially useful targeted agents is by co-administration of anticancer agents in association to pharmacological P-gp inhibitors.

Recently, we reported for the first time that morphine, conventionally used in the management of oncological patients, is a selective P-gp inhibitor [94]. Our group thoroughly investigated the ability of morphine to improve the accumulation and effectiveness of chemotherapeutic agents such as Dox in preclinical glioblastoma models.

Dox exhibits high in vitro cytotoxicity against primary glioblastoma cells [95], but P-gp actively extrudes the anthracycline at BBB level, limiting its effectiveness in the treatment of CNS tumors.

Combinational studies with morphine and Dox have been done in an in vitro model of BBB, to test the effect of the opioid agent on the intracellular accumulation of the anthracycline. Cytofluorimetric results have showed that morphine is able to increment Dox accumulation in P-gp transfected MDCKII cells [96]. For in vivo delivery evaluation, rats were pre-treated with morphine and other drugs, such as dexamethasone or ondansetron, before injection of Dox, and quantitative analysis of Dox levels was performed by LC-MS/MS. Data indicate that pretreatments with these agents allowed Dox accumulation inside all brain areas, including cerebral hemispheres, cerebellum and brainstem. Conversely, morphine administration was not associated with an augmented Dox accumulation in plasma, heart and kidney 1 h after treatment administration [97]. More interestingly, this combinational therapy was tested on a xenograft mouse model of glioblastoma. In vivo data reported that high Dox administrations, with or without morphine, resulted in up to 87% tumor volume inhibition, but this outcome was associated with undesirable side effects such as body weight loss. In contrast, low Dox *plus* morphine treatment showed an antitumor effect similar to high Dox administration, without animals’ body weight loss; rather, it induced a body weight gain of +7.98% as a clear signal of the treatment’s minimal toxicity [96].

Other groups have also evaluated combinational therapy to improve the efficacy of TMZ-based treatments [98,99,100].

TMZ is the standard of care for newly diagnosed glioblastoma patients, but its concentration in the brain is only 17–20% of the blood levels [101,102], and TMZ’s dose-limiting toxicity precludes the use of higher doses, which could theoretically result in higher intratumoral concentrations.

In 2011, Carman et al. demonstrated that regadenoson, an adenosine receptor agonist, increased BBB permeability to dextran (70 kD) in both mice and rat models [103].

Based on this evidence, Jackson et al. investigated the effect of regadenoson on TMZ accumulation in normal rodent brain. The animals received TMZ administration followed by a single dose of intravenous regadenoson. Brain and plasma TMZ concentrations were determined 120 and 360 min after the treatments using HPLC/MS/MS. After 120 min, TMZ concentration was 60% higher when it was given in association with regadenoson. Conversely, brain concentrations and brain plasma ratios were not significantly different 360 min after TMZ administration with or without regadenoson, indicating that the effect of the adenosine receptor agonist is reversible in a short time [104].

Our group has further investigated the potential of combined morphine *plus* TMZ treatments in glioblastoma therapy. Nude mice bearing human glioblastoma were treated with metronomic doses of TMZ in association with morphine. At the end of the treatment, the bioluminescence tumor signal of the TMZ *plus* morphine group showed a 2.5-fold lower value than that detected in the TMZ group. Moreover, this improved antitumor activity of the co-treatment was not associated to an increased systemic toxicity, evaluated in terms of body weight loss between the co-treated group and all other groups. However, we also demonstrated that the combination with morphine made possible a reduction in the TMZ dosage, with additional benefits in terms of efficacy and side effects. The co-treatment with lower TMZ *plus* morphine determined a tumor growth inhibition, with a tumor reduction of between 84% and 88% vs the control group. We also observed that while the effect of lower TMZ alone ended after the end of treatment, the combination of lower TMZ *plus* morphine maintained a more or less stable reduction from the end of the treatment until day +84, with a tumor reduction of 97.3% [94].

## 4. Genetic Aspects of Chemoresistance in Glioblastoma

Glioblastoma presents an infiltrative nature with genetic and cellular heterogeneity; in particular, it contains CSCs that are responsible for radio/chemotherapy resistance and tumor relapse, and have specific signatures [105,106,107,108]. Indeed, CSCs show increased expression of drug efflux ABC transporters, especially P-gp, MRP1 and breast cancer resistance protein (BCRP/ABCG2) [109], strong DNA damage response [110,111,112,113,114,115], reduced apoptosis mechanisms and altered regulation of transcriptional machineries. 

ABC transporters are membrane glycoproteins that, through ATP hydrolysis, extrude endogenous compounds and xenobiotics out of the cell. They have a physiological role in the transport of many types of molecules and an important protective function in the maintaining of the isolation of body compartments such as BBB and the blood-testis barrier [116]. The human ABC protein family is composed of 48 membrane transporter proteins [117] classified into 7 subfamilies (class A to G). To date, only P-gp, MRP1 and BCRP have been associated with MDR [118]. ABC transporters have broad substrate specificity in transporting the principal chemotherapeutic agents, and several non-responder cancers show a high expression of these transporters, determining a decreased therapeutic drug accumulation in tumor cells; in particular, P-gp-mediated MDR is regulated by various transcription factors and/or signaling pathways [119,120,121,122,123,124,125,126,127,128,129].

Another genetic mechanism involved in MDR is represented by alteration of DNA repair mechanism (DNA damage response, DDR). DDR is involved in the maintenance of genetic stability; in cancer cells, it can participate in the removal of DNA lesions induced by genotoxic anti-cancer agents and contributes to the development of chemoresistance and tumor relapse.

Currently, ionizing radiation (IR) and TMZ are the principal first line chemotherapy for the treatment of glioblastoma and induce the main DNA lesions. IR induces double-strand breaks (DSBs) repaired by homologous recombination (HR) and non-homologous end joining (NHEJ) mechanisms, and a base damage or single-strand breaks (SSBs) repaired by base excision repair (BER) and SSBs repair mechanisms, respectively. TMZ methylates DNA at the O6 and N7 positions of guanine and the N3 position of adenine [130]. N7-methylguanine and N3-methyladenine are repaired by BER and O6-methylguanine are repaired by O6-methylguanine DNA methyltransferase (MGMT) [131,132].

MGMT promoter methylation is reported in 30–60% of patients with glioblastoma [133,134] and is associated with an increased sensitivity to TMZ, a better clinical response to TMZ and a prolonged survival [134,135,136]. In absence of MGMT, the unrepaired 06-meG can incorrectly pair with thymidine; the mispairing activates the mismatch repair system (MMR), inducing DSBs through the “futile cycle” with consequent activation of specific signaling pathways of cycle arrest and cell death [137].

Defects in MMR are associated with TMZ resistance of glioma cells [138,139] and chronic exposure to TMZ can produce resistant clones harboring MSH6 mutations [139]. Indeed, the integrity of MSH2/MSH6 dimer is fundamental to induction of cytotoxicity in TMZ treatment. Glioma treated with TMZ acquires somatic MSH6 mutations, conferring TMZ resistance and resulting in a hypermutational process which supports rapid evolution of clones with growth advantage [140].

MicroRNAs (miRNAs) also play an important role in chemoresistance phenomenon of glioblastoma, given their versatile and ubiquitous nature. miRNAs, short endogenous single-stranded non-coding RNAs molecules, are a novel class of gene regulators involved in proliferation, control of cell differentiation, apoptosis, anti-viral defense; a deregulation of their expression has been reported in various tumor types [141,142,143,144]. However, they have a fundamental role in tumor progression and invasion [145] and several studies have demonstrated that miRNAs modulate drug sensitivity/resistance of various types of tumor included glioblastoma [146,147,148,149,150,151,152,153,154].

Regarding acquired TMZ resistance, it is known that this phenomenon is mainly modulated by high levels of MGMT protein [152,153,154,155,156,157], somatic inactivation of *MMR* genes [158], and high expression level of the BER system [159,160]; literature data report that both glioblastoma cell lines and glioblastoma patients with acquired TMZ resistance show a specific miRNAs expression profile [161,162]. These findings support the hypothesis that miRNAs have a remarkable supporting role in the main mechanisms involved in acquired TMZ resistance. 

miRNAs are also implicated in MDR through upregulation of the expression of drug transporter genes in glioblastoma and other tumors [163,164,165] or through modulation of genes and/or specific signal pathways implicated in ABCB1/P-gp-mediated MDR [166] or regulation of specific signal pathways involved in repair of cellular damage caused by cytotoxic therapy and in cell proliferation [167,168,169,170].

Finally, miRNAs can promote oncogenic transformation or perform their function as modulators of chemoresistance through their release into exosomes. Exosomes are small extracellular vesicles and contain DNA, RNAs, MicroRNAs, growth factors and enzymes released from tumors; these vesicles play a role in cell-cell communication and are involved in important physiological and pathological processes [171,172,173].

## 5. Immunotherapy in Glioblastoma: Overcoming Barriers to Response 

Patients with glioblastoma have a poor survival rate despite aggressive treatments such as surgical resection, radiation and chemotherapy. Furthermore, these current therapies have an immunosuppressive effect, and they have no effect at recurrence [174].

Considering that all patients with glioblastoma have disease relapse, the approaches used at tumor recurrence or progression necessarily have to be more individualized. The lack of second line therapy is highlighted by the fact that a substantial proportion of patients (around 50%) does not receive any therapeutic intervention at the time of progression, meaning that evidence is lacking that secondary approaches have effects in terms of survival [175,176]. Therefore, different immunotherapy approaches have to be investigated, because the brain’s microenvironment may offer the opportunity to implement treatment.

The concept that CNS is an immune privileged system is obsolete today [177]; in fact, Medawar’s group has demonstrated that engraftment of foreign cells in the brain of rodents was prevented by vaccination of the animals against the same foreign cells before the implantation [178,179,180].

Moreover, it has been described that a lymphatic egress runs parallel to dural venous sinuses, and that antigen-presenting cells could travel to deep cervical lymph nodes. According to these concepts, the immunotherapy approaches could bypass the obstacle of BBB. The activated T cells could be primed against tumor-specific antigens and traverse the BBB through adhesion markers (VLA-4), allowing them to penetrate the TME [181].

Glioblastoma is surrounded by an immunosuppressive microenvironment. Tumor cells express increased level of immunosuppressive factors, such as programmed cell death protein-1 (PD-1), indolamine 2, 3 dioxygenase (IDO), STAT 3 and FASL. Furthermore, microglia cells (considered to be the major antigen presenting cells in the brain) secrete TGF-B and IL-1 that downregulate the local myeloid and lymphatic immune cells and promote systemic immunosuppression [182].

Treg cells contribute to the immunomodulating environment through upregulation of several immune-check-point molecules (IL-10, denosina, LAG3, CTLA-4); and myeloid cells, including TAMs, have an immunosuppressive and tumor-promoting effect, modifying the expression of various extracellular and intracellular mediators [183]. All these factors modify the phenotype of cytotoxic T lymphocytes (CTLs), increasing the levels of markers such as PD-1. Dendritic cells (DCs) can run along deep cervical lymph nodes and present antigen to promote an adaptive antitumor immune response [184]. These mechanisms are still unknown but, starting from these concepts, several studies have been developed to encourage antitumor immune responses. The main therapy could be the use of check point inhibitors, which have to be combined with target therapy [185]. The immune checkpoint blockade antagonizes inhibitor receptor/ligand interaction with monoclonal antibodies, which allows activated T cells to mediate their antitumor effector. The capacity of the tumor cells to evade destruction by CTLs could be bypassed by treatment with anti-PD-1 and anti-CTLA-4 antibodies, reinforcing the adaptive arm of the immune system. Furthermore, using vaccine therapies, a tumor’s antigens, peptides or epitopes are presented to DCs. The CTLs are subsequently activated to destroy tumor cells containing glioblastoma-associated antigens such as IL-13Rα2 or EGFRvIII. There are some clinical trials of therapeutic vaccine for glioblastoma that use these vaccines *plus* standard treatment such as TMZ, bevacizumab and radiation therapy [186]. To date, there are no significant results in terms of overall survival between treatment with immunotherapy and standard approaches [187].

Another therapy might be an oncolytic viral therapy to create a virus that could activate the immune system. Oncolytic viruses are attenuated to propagate in tumor cells. In fact, tumor cells lack a viral defense mechanism that prevents viral DNA integration into their genome until they are recognized by the immune system for rejection. However, few data showing preliminary efficacy are currently available [188].

**Figure 3:**
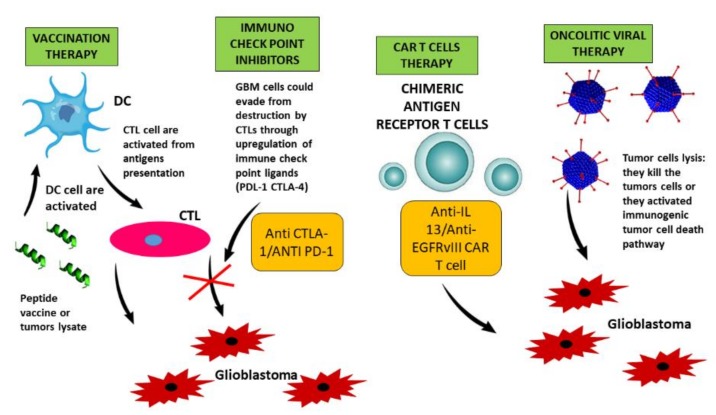
Glioblastoma vaccine consists of the presentation of glioblastoma-associated antigens, peptides or epitopes (derived from tumor lysate to T cells). The tumor cells could evade the destruction by CTLs through the increase of immune checkpoint ligands. The use of checkpoint inhibitors could prevent this interaction. The presence on the glioblastoma cell surface of IL-13 and EGFR receptors, which are presented independently of MHC class I, makes it possible to develop modified CAR T cells. Oncolytic viral therapy uses a virus that can activate the immunogenic cancer cell death pathway or kill the tumor cell directly.

Cancer vaccines and oncolytic viral therapy try to stimulate an innate immune response and to produce a successful organization of immune stimulus. Glioblastoma-associated antigens, including IL-13 receptor subunit-α2 (IL-13Rα2) and EGFR variant III (EGFRvIII), are also presented on tumor cell surfaces independent of major histocompatibility complex (MHC) class I, a necessary requirement for T cell recognition. With engineered chimeric antigen receptor (CAR)-modified T cells, MCH downregulation can be overcome by tumor cells [189]. They can recognize antigens that are not presented in the context of MHC molecules, as typically required for adaptive immune response. Moreover, they can identify and reject tumor antigens such as HER-2 or IL13Rα2 [190] (Figure 3).

However, this approach remains limited because CAR T cell targeting of normal antigens in the brain may lead to CNS neurotoxicity, inflammation, increased intracranial pressure and herniation [191].

Interestingly, these treatments could be associated with liquid biopsy to analyze the genetic makeup of the brain [192,193]. Even though tumor-derived DNA and circulating tumor cells are rarely detectable in the blood of patients with glioblastoma, the sample could be analyzed to select appropriate patients for immunotherapy [194].

Although no excellent results have been obtained yet, future immune-based strategies are focused combining standard treatment and different immunotherapy, in particular checkpoint inhibitors. This field could be implemented to become a part of standard of care for patients with glioblastoma.

## 6. Conclusions

The phenomenon of chemoresistance developed in glioblastoma to conventional treatments is related to its biological complexity and to different cellular and molecular mechanisms activated during tumorigenesis and progression. Research into the dynamic interaction between microenvironment and cancer cells is rich with potential for improving treatment of this lethal tumor. BBB is crucial to maintaining a stable microenvironment, but it also represents the major hurdle for the delivery of antineoplastic agents into the brain. Many aspects of glioblastoma chemoresistance have been reviewed here, including mechanisms of chemoresistance due to changes in tumor microenvironment, pharmacological modulation of BBB, genetic aspects of chemoresistance and the role of immunotherapy, but further original research is needed to make a significant impact for patients with glioblastoma.

## Abbreviations

ABC	ATP binding cassette
ABCG2	ATP binding cassette subfamily G member 2
ADT	Ala-Asp-Thr
BBB	blood brain barrier
BCRP	breast cancer resistance protein
BER	base excision repair
CAFs	cancer-associated fibroblasts
CAR	chimeric antigen receptor
CNS	central nervous system
CSCs	cancer stem cells
CTLs	cytotoxic T lymphocytes
DCs	dendritic cells
DDR	DNA damage
Dox	doxorubicin
DSBs	double-strand breaks
ECM	extracellular matrix
EGFRvIII	EGFR variant III
GdDTPA	gadolinium-DTPA
GSCs	glioblastoma stem cells
HAV	His-Ala-Val
HIFs	hypoxia-inducible factors
HR	homologous recombination
IDO	indolamine 2,3 dioxygenase
IL-13Rα2	IL-13 receptor subunit-α2
IR	ionizing radiation
MDCK	madin-darby canine kidney
MDR	multi drug resistance
MDR1	multi drug resistance 1
MDSCs	myeloid-derived suppressor cells
MGMT	O6-methylguanine DNA methyltransferase
MHC	major histocompatibility complex
miRNAs	microRNAs
MMR	mismatch repair system
MRP1	multi drug resistance-associated protein 1
mTOR	mammalian target of rapamycin
NDRG1	N-myc downstream regulated gene 1
NHEJ	non-homologous end joining
NK	natural killer
PD-1	programmed cell death protein-1
PEG	polyethylene glycol
P-gp	P-glycoprotein
R800	rhodamine 800
ROS	reactive oxygen species
SSBs	single-strand breaks
TAMs	tumor-associated macrophages
TEER	transepithelial electrical resistance
TME	tumor microenvironment
TMZ	temozolomide
Treg	T-regulatory
VEGF	vascular endothelial growth factor
